# The Evaluation of Virtual Reality Neuroanatomical Training Utilizing Photorealistic 3D Models in Limited Body Donation Program Settings

**DOI:** 10.7759/cureus.55377

**Published:** 2024-03-02

**Authors:** Martin Trandzhiev, Theodoros Koundouras, Milko Milev, Lili Laleva, Atanas Mitev, Viktor Stoykov, Nikolay Dimitrov, Ivan Maslarski, Vladimir Nakov, Toma Spiriev

**Affiliations:** 1 Department of Neurosurgery, Acibadem City Clinic University Hospital Tokuda, Sofia, BGR; 2 Department of Anatomy and Histology, Pathology and Forensic Medicine, University Hospital Lozenetz, Medical Faculty, Sofia University, Sofia, BGR

**Keywords:** photogrammetry, education, photorealistic three-dimensional models, virtual reality, neuroanatomy

## Abstract

Background

Neuroanatomy is one of the most complex areas of anatomy to teach to medical students. Traditional study methods such as atlases and textbooks are mandatory but require significant effort to conceptualize the three-dimensional (3D) aspects of the neuroanatomical regions of interest.

Objectives

To test the feasibility of human anatomy teaching medical students in a virtual reality (VR) immersive environment using photorealistic three-dimensional models (PR3DM) of human anatomy, in a limited anatomical body donation program.

Methods

We used surface scanning technology (photogrammetry) to create PR3DM of brain dissections. The 3D models were uploaded to VR headsets and used in immersive environment classes to teach second-year medical students. Twenty-eight medical students (mean age 20.11, SD 1.42), among which 19 females (n=28/67.9%) and nine males (n=28/32.1%), participated in the study. The students had either none or minimal experience with the use of VR devices. The duration of the study was three months. After completing the curriculum, a survey was done to examine the results.

Results

The average rating of the students for their overall experience with the method is 4.57/5 (SD=0.63). The “Possibility to study models from many points of view” and “Good Visualization of the models” were the most agreed upon advantages, with 24 students (n=28, 85.7%), and 95% confidence intervals (CI) [0.6643, 0.9532]. The limited availability of the VR headsets was the major disadvantage as perceived by the students, with 11 students (n=28, 39.3%), 95% CI [0.2213, 0.5927] having voted for the option. The majority of the students (25) (n=28, 89.2%, SD=0.31) agreed with the statement that the use of VR facilitated their neuroanatomy education.

Conclusion

This study shows the future potential of this model of training in limited cadaver dissection options to provide students with modern technological methods of training. Our first results indicate a prominent level of student satisfaction from VR training with minimum negative reactions to the nature of headsets. The proof of concept for the application of photorealistic models in VR neuroanatomy training combined with the initial results of appreciation among the students predisposes the application of the method on a larger scale, adding a nuance to the traditional anatomy training methods. The low number of headsets used in the study limits the generalization of the results but offers possibilities for future perspectives of research.

## Introduction

Neuroanatomy has always been one of the most challenging subjects to study and teach in the medical curriculum. The reason behind this is the complex microscopic elements and their interactions, as well as the difficulties of presenting these structures without special fixation methods [1,2].

Traditional anatomical dissections are widely appreciated by medical students, but such classes are limited to a specific timeframe outside of which the students should adhere to classical study methods such as atlases and textbooks [3]. The obstacles that arise throughout the entire process aid students' potential, but at the same time may increase prejudices and reinforce neurophobia [2].

On the other hand, the steep improvement of innovative technologies such as virtual reality (VR), augmented reality (AR), and photorealistic surface scanning significantly impacts medical education [1]. The inflow of evolved interactive tools generates a better study environment, by facilitating teachers’ efforts and maximizing students’ potential at the same time [4].

Notably, the development of photorealistic three-dimensional models (PR3DM) and their integration into medical education has amounted to an impact on the involvement and enjoyment of the study process due to the photorealistic nature of the data used [5,6]. The effectiveness of PR3DM as a study tool has its origin in the technique of photogrammetry, a well-established technique that can be used to generate 3D models in the gaming industry and has only recently been applied to produce visualizations of cadaveric specimens for anatomical studies [7-10].

Together with new advancing technology such as VR systems, comprising head-mounted displays with tracking systems, navigation tools, and sound to ensure a maximal sense of immersion, PR3DM’s integration can promote active learning processes in different fields through immersive experiences in virtual environments, and in the same time provide opportunities for scientific research [11-13].

Therefore, the utilization of PR3DM and their integration into a VR environment has the potential to facilitate the neuroanatomy educational process by creating a more engaging form of study, independent of cadaver specimen training [14,15].

In our study, we used the photogrammetry method to scan neuroanatomy dissection models - (brain, peripheral nerves, cranial bone structures) with apparent preservation of geometry and texture with maximal similarity to the original specimen. We aim to evaluate the potential benefits and disadvantages of neuroanatomical PR3DM with annotations in immersive VR for teaching medical students in an environment with limited cadaver specimen exposure.

There are multiple ethical and legal factors that limit body donation programs and consequently exposure to anatomical training. Alternatives must be created in order to ensure adequate anatomical medical education. Therefore, we conducted a study using modern technologies which allowed us to create a photorealistic database of 3D models of actual neuroanatomical specimens, which were applied in a VR environment in order to supplement anatomy studying.

## Materials and methods

Study design and settings

This prospective cohort study (duration: May 2023 to August 2023) included second-year medical students, undergoing a series of VR classes, with a survey for the evaluation of their experience with the method. The study included only the students from the classes of the senior author since the number of VR headsets was limited (three headsets for the whole duration of the study), as well as the number of faculty staff acquainted with the VR technology and 3D modeling was also limited. The study received ethical approval as part of project number 80-10-182/17, May 2023, from the Sofia University, Faculty of Medicine.

Twenty-eight medical students (mean age 20.11, SD 1.42), among which 19 females (n=28/67.9%) and nine males (n=28/32.1%), took part in a series of classes during which they benefited both from conventional anatomy learning methods, such as two-dimensional (2D) atlases and lectures as well as 3D models in the immersive VR environment. The neuroanatomy classes were conducted as follows - half of the time of the class the students had VR sessions with dedicated neuroanatomical models for the current subject (brain cortex, subcortical white matter, brainstem, cerebellum, etc.) and the other half the students had to examine already dissected real physical anatomical specimens and identify anatomical structures of interest. The students had the opportunity to study the models and their annotated structures from inside the immersive VR environment, where each of the models could be freely manipulated. A total of seven questions evaluated empirical data and five questions evaluated non-empirical data. The questionnaire aimed to assess three main factors: (1) the overall experience of the students with the method and their perceived benefit from using it; (2) the comparison of the method with the conventional anatomy teaching methods; (3) the advantages and disadvantages of the method. Mean, standard deviation and 95% confidence intervals were calculated for the answers of the students. A survey with 12 questions was created on Google Forms and conducted to evaluate the experience with the new method of learning as well as its advantages in comparison with the conventional methods, as perceived by the students. The questionnaire was not validated, developed specifically for the occasion, and was anonymous. All of the students consent prior to participating in the study. The types of questions included in the survey were designed to collect both empirical and non-empirical data (see Appendices). Descriptive statistic was done on the results, including mean, standard deviation, and CI on Microsoft™ Excel™ 365 MSO 64bit (Microsoft Corporation, Redmond, Washington, USA).

Model generation

Human anatomy PR3DM was created based on dissected cadaver specimens through the photogrammetry surface scanning technique. For the study, different anatomical regions and organs were dissected and scanned - brain sequential dissections and whole brain specimens; topographic neck region dissections to present cranial nerves; back muscles layered dissection to present the course of the accessory nerve and brachial plexus nerves. As a result, a large number of neuroanatomical 3D models were created for use during the study. The generation of the models followed a simple methodological pipeline, which included image acquisition with a digital single-lens reflex (DSLR) camera or a smartphone, and later model creation and model editing with dedicated software. We used a smartphone camera (iPhone 11, Apple Inc., CA, USA) combined with the Metascan (Abound Labs Inc., New York, USA) software, to generate the data [15,16].

Virtual reality

**Figure 1:**
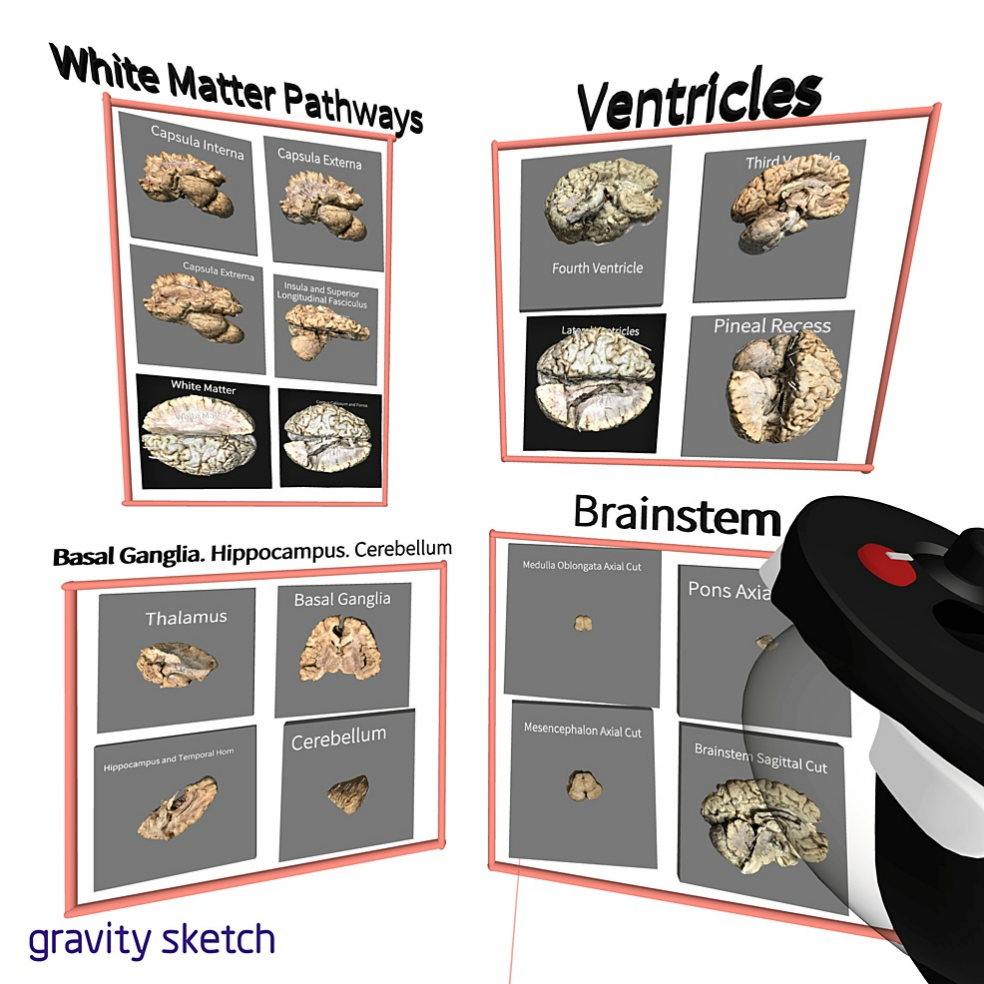
Photorealistic three-dimensional model collections in an immersive virtual reality environment The figure is a screenshot from the VR headset inside the immersive VR environment. Four clusters of models presenting different anatomical components of the central nervous system inside the Gravity Sketch platform. The models can be “picked up” and manipulated in the immersive space using the joystick of the Oculus headset.

For the study, the models were uploaded to the free (at the time of manuscript preparation) VR 3D modeling platform Gravity Sketch (https://www.gravitysketch.com/), where they were divided into different clusters for the presentation of different anatomical segments (brain, neck, back muscles). The brain dissection models were additionally subdivided into four groups inside the immersive environment - "White Matter Pathways", "Ventricles", "Basal Ganglia, Hippocampus, Cerebellum", and "Brainstem" (Figure 1).

**Figure 2:**
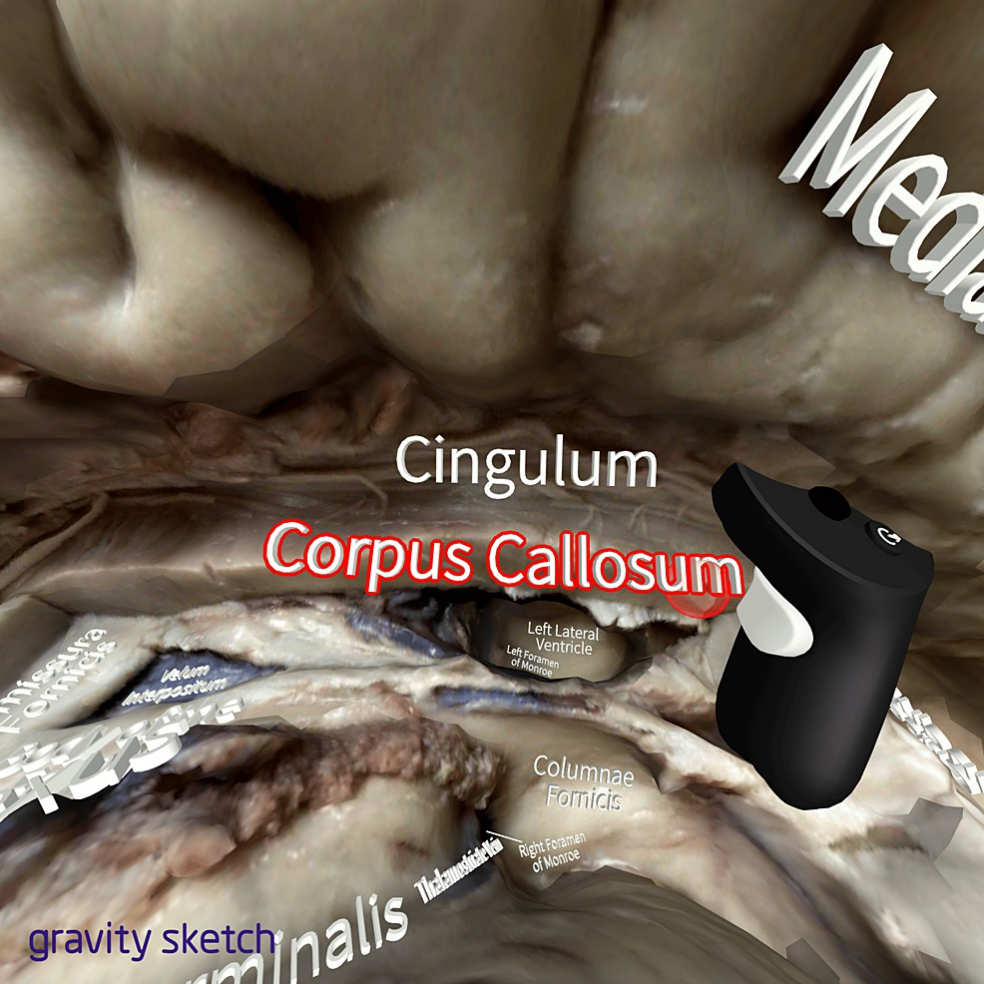
Annotation of a model The figure is a screenshot from the work in the VR environment. The annotations of the anatomical structures of the models through the use of the tools in the Gravity Sketch platform. The text “Corpus Callosum” is being put and stuck on the correct part of the model. The red contour around the text "Corpus Callosum" indicates that the text, as an object, is currently held with the right controller of the user. The adjacent structures (e.g., Lateral Ventricle, Cingulum) have also been annotated through the same method.

The specific structures of the models were additionally annotated using the properties of the same platform (Figure 2).

**Figure 3:**
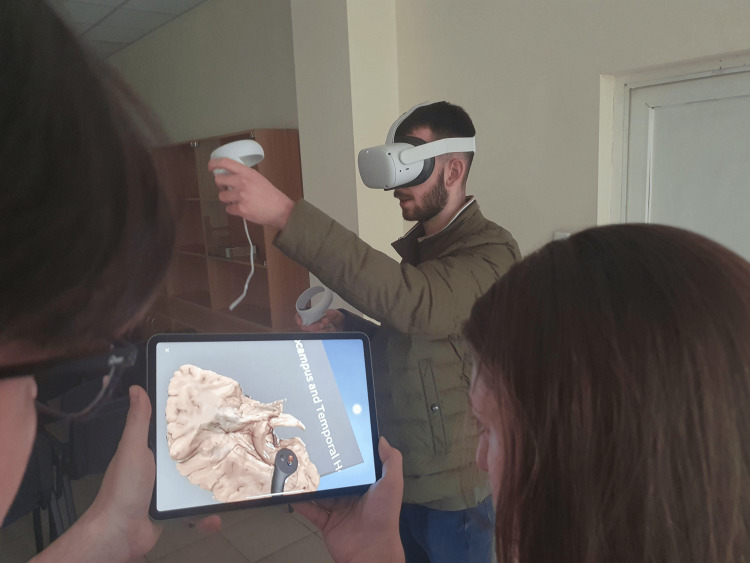
Students observing the work in the immersive environment The connection between the Oculus headset and the iPad through a dedicated application allows the students to observe on the iPad screen in 2D everything that the user of the headset is seeing in the immersive environment.

The Oculus Quest 2 (Meta Platforms Inc., Menlo Park, USA) headset was used for the VR part of the study. The VR headsets were connected to a tablet which displayed the experience of the user inside the VR environment so that the students could rotate between using the headset to work with the models and observing the work of the others (Figure 3).

**Figure 4:**
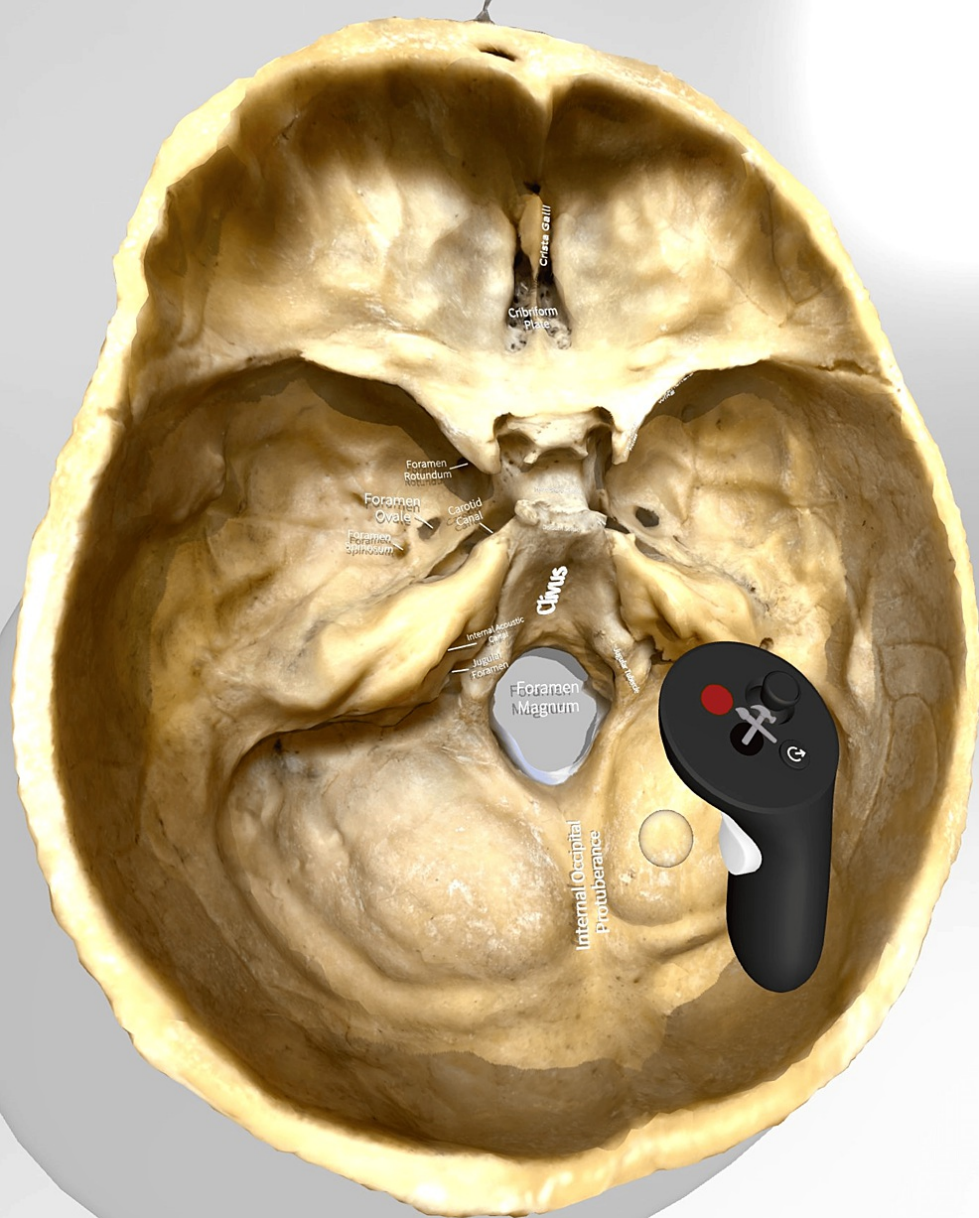
Model of the skull base Model showing the skull base in the immersive environment. The detail of the model is preserved when uploaded on the platform and it can be manipulated in numerous manners: changing the model’s size, moving the model around, rotating the model, zooming in and out of important structures, and annotating the model.

Thus, a wide variety of means of observing the 3D models was available. Neuroanatomical models were used, such as models of the brain (hemispheres, basal ganglia, brain vasculature, cranial nerves, ventricles, brainstem) (Figure 1) and skull (skull bones, skull base) (Figure 4).

Models of the anterior neck region and back muscles were also included.

## Results

Twenty-eight answers were collected from the students at the end of the study. The data derived from the answers were divided into empirical and non-empirical, based on the type of question and information collected.

Empirical data analysis

**Figure 5:**
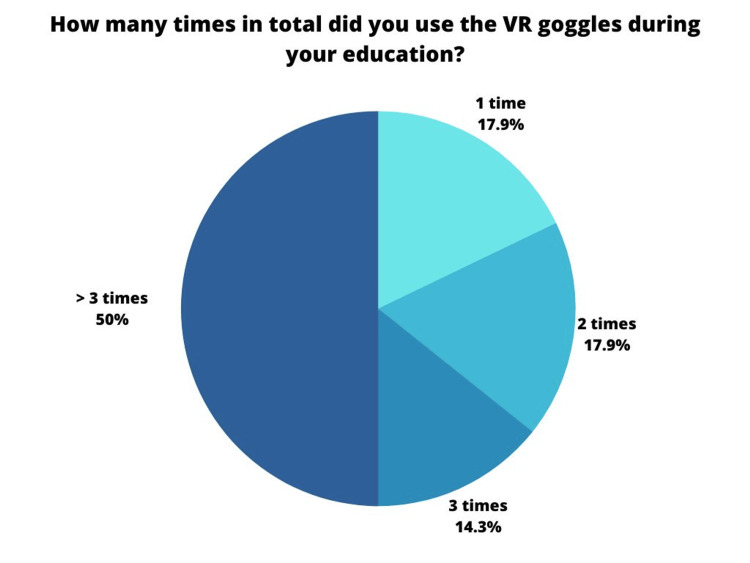
Question number 1 Fourteen students (50%) had the chance to participate in the exercises more than three times, four students (14.3%) participated exactly three times, five students (17.9%) participated two times, and five students (17.9%) participated only one time.

Half of the students had the opportunity to participate in the VR classes more than three times and an additional 14.3% participated a total of three times, which shows a high level of engagement among the students, participation was optional (Figure 5).

**Figure 6:**
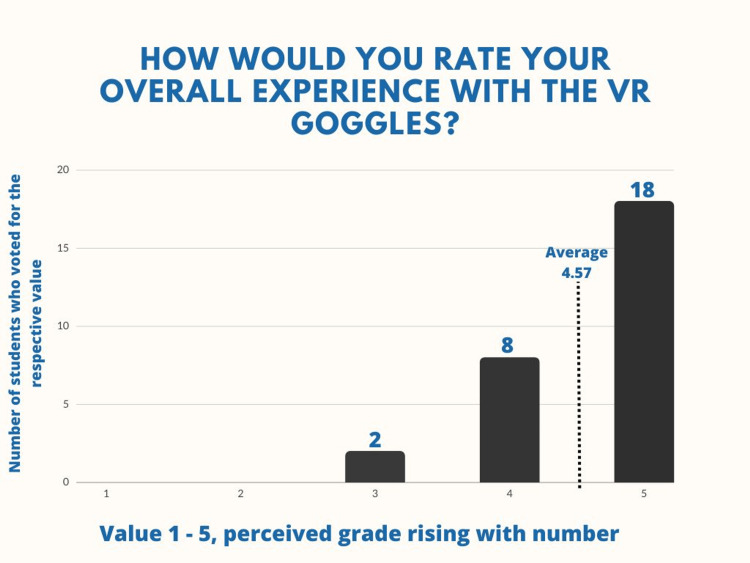
Question number 2 When asked to rate their overall experience with the VR exercises on a scale from one to five, 18 students rated it with a 5/5, eight students rated it 4/5, and two students rated it 3/5 (M=4.57; SD=0.63).

The VR method is well accepted by the students, taking into account the fact that when asked to rate their overall experience, the students had a mean score of 4.57 out of 5 which points to a high level of satisfaction (Figure 6).

**Figure 7:**
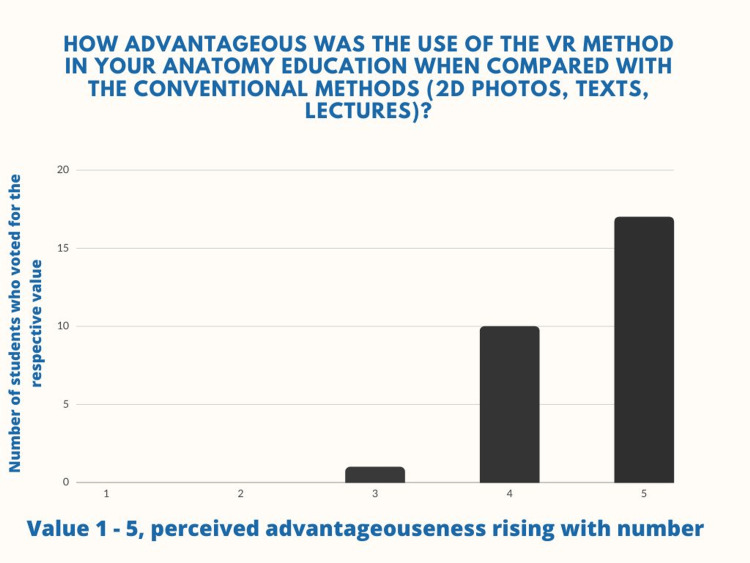
Question number 3 Seventeen (60.7%) of the students rated the VR method as very advantageous when compared to the conventional methods, ten (35.7%) rated it as moderately advantageous, and one (3.6%) student rated it as neither advantageous nor disadvantageous (M=4.57, SD=0.57).

Additionally, 96.4% of students agreed that the method is either significant or moderately advantageous when compared with the conventional methods of anatomy teaching and only one student voted that he was unsure on the matter, with neither having rated the novel method as disadvantageous (Figure 7).

**Figure 8:**
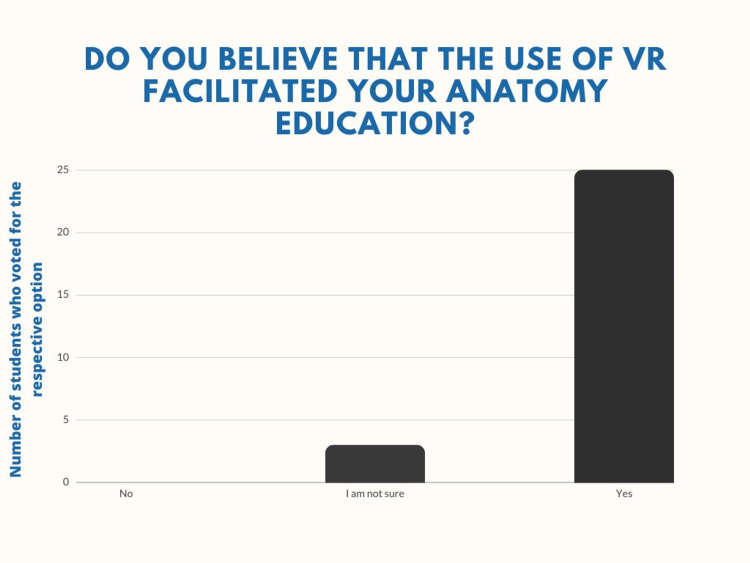
Question number 4 Twenty-five students (89.3%) agree with the statement, that the VR method facilitated their anatomy education, while three students (10.7%) are unsure of the matter (M=1.11, SD=0.31). Neither of the students expressed disagreement with the statement.

A total of 89.2% agreed that the method facilitated their neuroanatomy education, with three students being unsure on the matter. Not a single student expressed disagreement with the statement (Figure 8).

**Figure 9:**
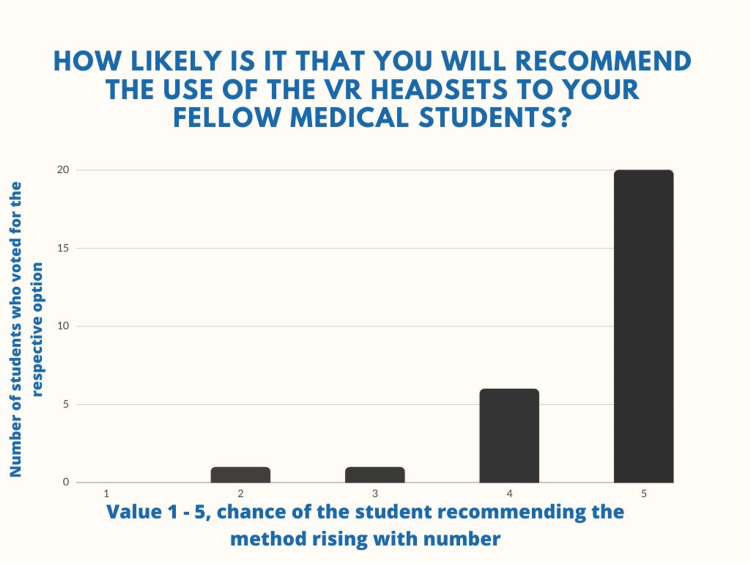
Question number 5 When asked how likely it is that they would recommend the VR method to their fellow students, 20 of the students (71.4%) voted that it is highly likely, six (21.4%) voted that it is moderately likely, one student (3.6%) voted that they are neutral on the matter and one student (3.6%) voted that it is unlikely (M=4.6, SD=0.74).

The method is also well-endorsed by the students - 92% are either highly or moderately likely to recommend it to their fellow colleagues (Figure 9).

**Figure 10:**
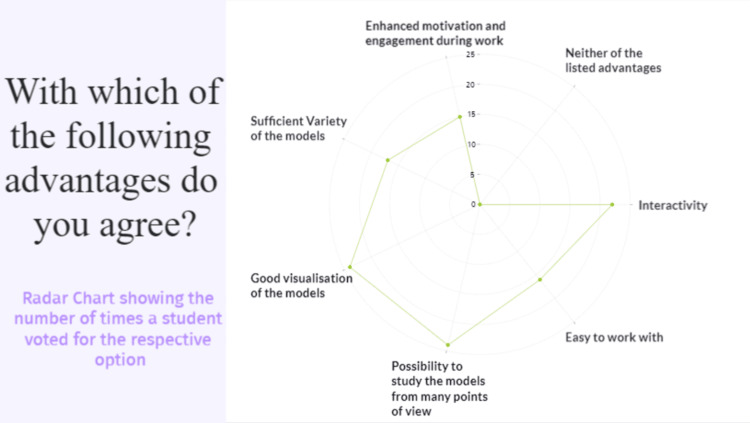
Question number 6 The advantages most agreed upon were the “Possibility to study the models from many points of view” and “Good visualization of the models”, with 24 students (85.7%) having voted for these options. “Interactivity” is second with 22 students (78.6%) agreeing upon that advantage, followed by “Sufficient variety of the models” with 17 votes (60.7%) and “Easy to work with” with 16 votes (57.1%). Fifteen (53.6%) of the students agreed with the advantage of “Enhanced motivation and engagement during work”. Neither of the students voted for the “Neither of the listed advantages” option.

According to the students, the “Possibility to study the models from many of view” and the “Good visualization of the models” are the major advantages of the method - with 85.7% of the students having voted for both of the options, with a 95% CI calculated at [0.6643, 0.9532]. The second most agreed upon advantage was the “Interactivity”, with 78.5% of the students, 95% CI [0.5854, 0.9097], followed by “Sufficient variety of the models” - agreed upon by 60.7%, 95% CI [0.4073, 0.7787], “Easy to work with” - agreed upon by 57.14%, 95% CI [0.3743, 0.7497], and “Enhanced motivation and engagement during work” - with 53.57% having voted for the option, 95% CI [0.3421, 0.7199]. It’s important to point out, that not a single student voted for the “Neither of the listed advantages" option, 95% CI [0, 0.1502] (Figure 10).

**Figure 11:**
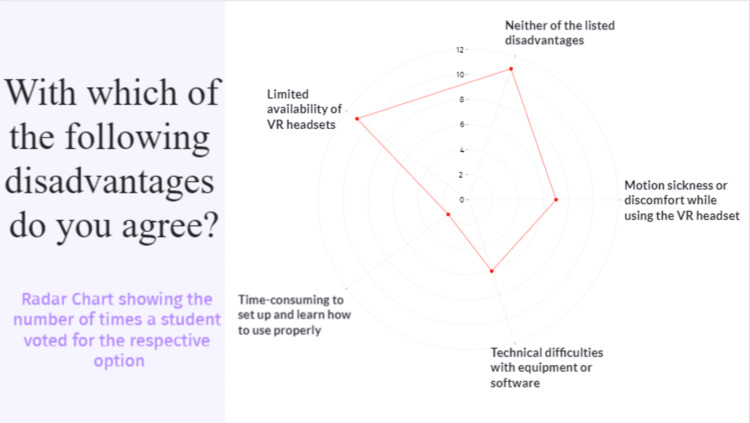
Question number 7 The disadvantage most agreed upon was “Limited availability of the headsets” with 11 (39.3%) votes. Seven students (25%) reported “Motion sickness or discomfort while using the VR headset” and six students (21.4%) reported “Technical difficulties with equipment or software” as their perceived disadvantages. Only two students (7.1%) found the method “Time-consuming to set up and learn how to use properly”. Eleven students (39.3%) voted for the “Neither of the listed disadvantages” option.

This shows how the advantages outweigh the disadvantages, seeing as 11 students (39.3%) voted for “Neither of the listed disadvantages” option, 95% CI [0.2213, 0.5927]. Nevertheless, the major identified disadvantage was the “Limited availability of the headsets”, with 39.3% of the students agreeing with the option, 95% CI [0.2213, 0.5927], which shows a key area of potential logistical improvement in the classes. The rest of the disadvantages were voted for by less than a quarter of the surveyed students (Figure 11).

Non-empirical data

Among the non-empirical data collected, we have identified certain answers which could be beneficial to the study. Nevertheless, this data is of lower relevance for the study than the empirical data.

Three students reported additional advantages, one of them claimed that the learning process of the models was facilitated in the VR environment, and two commented on the spatial orientation provided by the method.

Four students reported additional disadvantages. The first student reported problems with visual accommodation in VR for people with eye conditions, such as myopia or hypermetropia. The second student reported getting a headache after the use of the VR headset. The third student reported misalignment of the annotations and the 3D model in the VR environment - this problem is easily fixed with the tools in the Gravity Sketch application. The fourth student reported the lack of descriptive text on the structures as a drawback.

## Discussion

The integration of PR3DM in the world of VR has the potential to change the field of medical education. Photogrammetry applied to neuroanatomy has led to the creation of authentic dissection-based 3D models with enhanced detail, that open new possibilities for teaching and learning neuroanatomy [9,10]. The incorporation of VR into the learning curriculum adds a multidimensional and immersive experience to the user for each area of the anatomical area of interest while enhancing, the interest and motivation to learn [14].

The objective of the study was to provide an opportunity for each medical student to learn through interaction with various dissection-based neuroanatomy PR3DMs, while at the same time receiving guidance and explanations from anatomists through the study process. In our study, 79% of the student sample voted “Interactivity” and 85% voted “Possibility to study the models from many points of view” as one of the main advantages of the VR method of education, which as other authors outline as well, provides a more intuitive and engaging form of study process involving more than one sense (visual as well as motor system) [11,17]. The study of Ekstrand et al. shows that when presented with a VR and paper format, two distinct groups of students do not have a statistically significant difference in test outcomes [11]. The authors proposed that future studies should include a longer follow-up of the work with the VR devices and the cost-benefit of the method should be taken into account [11]. Furthermore, they highlighted two aspects that our study showcases as well, namely that a different level of motivation was likely experienced by the VR group, as well as the fact that a learning curve is associated with the employment of the devices in practice [11,17]. Aridan et al. conducted a study with a similar methodology to ours and presented the results through a survey, which showed that spatial methods of learning, such as VR, were highly appreciated by the students [17]. One of the advantages of our methodology is the use of the Gravity Sketch platform, which allows us to directly annotate structures of interest on the models without the need to employ images of the models with the abbreviated structures for indication.

The concept of learning styles between different people demonstrates that there are major differences in the means, time employed, and success rate in learning certain subjects [18]. Statistically significant differences were found between students for the amount of time required as well as the techniques involved in studying [18]. The exact relationship between learning in a VR environment with PR3DMs and the four different learning styles (divergent, convergent, assimilative, accommodative), as well as the contrast in the same aspect with the conventional methods, has not yet been studied. Future directions could expand more on the amount of time required for learning a subject with the help of a VR teaching aid.

There are conflicting literature data regarding the advantages of VR-based learning compared to conventional methods [14]. According to Stepan et al., there are no statistically significant findings to prove performance differences (in validated examination results) in student training using VR environment to traditional forms of 2D study [13]. However, the study also highlighted the importance of the sense of presence - the subjective feeling of immersion that VR provides, and its correlation with the performance of the participants [13]. Consequently, having even more advanced immersive environments that enhance the sense of presence has the potential to bring improvement in the scores, since both the interactivity, the visualization of the models, and the participants' subjective motivation can be positively impacted [11,13,15]. This hypothesis is in accordance with our results since 15 of the students agreed with the “Enhanced motivation and engagement during work” advantage of the VR method, accounted for mostly by the novel and immersive nature of the devices, offering a different incentive to study. Furthermore, other authors provide meta-analysis data from 15 randomized control studies using VR as a teaching method, that the latter is an efficient method to improve students’ test scores in anatomy compared to those who were trained by traditional teaching methods [19]. In our study we have not directly evaluated and compared outcomes of student test results. However, 89% of the students highlighted our method as a means that facilitates the learning process, which indicates that certain aspects of VR training have impacted their efforts in a different manner than the conventional methods [18]. Unequivocally, more than 90% would recommend it to their colleagues, thus showing the value of the new VR teaching approach, which makes the whole study process of difficult subjects such as neuroanatomy more intriguing and interactive to students.

Fundamentally, the creation of PR3DM which was used for the accomplishment of our VR neuroanatomy training is based on the science of photogrammetry, which constitutes the creation of 3D models, later to be annotated [9,10]. In our study, we used a simple and optimized photogrammetry method described in detail elsewhere [10,15,16]. We chose this method as it can give optimal precision in dimensions and quality in 3D texture in comparison with more expensive surface light scanners or laser 3D scanning [7]. This allowed us to create a more thorough database of brain models, which is noted in the questionnaire as the “Sufficient variety of models,” an option for which 17 students voted.

The principal benefit of photogrammetry is the creation of realistic 3D models since they are generated by photographs, which translates to materials and texture qualities consistent with the actual cadaveric specimens [6,9,10,15,16]. This optimization allowed us to augment the photorealistic database used as a basis for VR training with further resemblance of the 3D models to actual anatomical structures, which has been reflected in the survey with the option “Good visualization of the models”, for which 85% of the students voted as an agreed-upon advantage. The photogrammetry process has been more widely applied for the creation of similar databases in recent years, as well as for diagnostic and other purposes in neurosurgery [8,20,21]. We believe that such a degree of realism and the immersive interactivity that VR provides is the basis of the positive feedback that we have from the medical student’s questionnaire. The additional annotations, which were added to the models through the use of the Gravity Sketch platform, allowed students to better navigate the models and spare time, which would normally be used by the teacher to name the structure of interest to the student. As far as we are aware, this is the first instance of the platform being used for this purpose. The use of the application through the Oculus Quest 2 headset offered a wireless experience with various tools for the manipulation of the models, allowing the user to enlarge the models of interest and look at them from different points of view [17,19].

Recently, Gonzalez-Romo et al. have presented the use of a collaborative VR environment, which allows multiple users to log into the same environment and study the same digital models from remote locations [22]. The method facilitates effective communication between remote departments on one hand, and on the other predisposes larger studies on the subject of neuroanatomy education in an immersive VR environment, since the geographical factor is less of an obstacle [22].

Limitations

Our study was conducted with a limited number of VR headsets with a limited number of participants which precludes the generalization of the results. A larger number of participants would increase the statistical potential and may provide a better understanding of the software difficulties during the training period. Furthermore, the reliability of these data is impacted by the limited number of available headsets. In any case, access to a greater number of devices would potentially provide better interpretation for each advantage, limitation, or disadvantage that emerges during the whole VR neuroanatomical training. The lack of assessment of the learning activity of the students is one of the main limitations of this study. For future studies, the students will be divided into two groups - one trained through conventional methods and the other with the use of the novel method, to evaluate and compare their results at an exam. However, this study reflects the reality of how for a smaller group of students, a VR neuroanatomical study teaching program can be initiated with a limited budget when PR3DM is readily available, since a lower number of VR devices is required. The initial positive results give us the motivation to perform future studies on the subject exploring the above-mentioned limitations.

## Conclusions

This study presents the preliminary results of our efforts to improve neuroanatomy teaching and studying conditions in an environment of limited availability of cadaver specimens and budget. The PR3DM has the potential to facilitate the learning of complex anatomical structures and the relationships between them through visualization in an immersive VR reality environment, as well as to lower neurophobia and enhance student motivation. The additional modifications applied to the models, such as the annotations in the Gravity Sketch platform, further augment the method and facilitate structure recognition.

Through our survey, we offer an internal validation of the students' impressions and experience with the method as well as the major advantages and general drawbacks as perceived by them. The analysis of the data has shown us the major strong points to emphasize in future applications of the method as well as the key drawbacks and limitations to improve.

## Appendices

**Table 1:** Questionnaire Table showing the questions which were used for conducting the survey, divided into empirical and non-empirical data. The available answers for the empirical data were provided.

Type of data	Questions	Answer options
Empirical	1. How would you rate your overall experience with the VR goggles?	Linear scale: (very bad) 1 - 2 - 3 - 4 - 5 (very good)
Empirical	2. How many times in total did you use the VR goggles during your education?	Single answer: 1. One time; 2. Two times; 3. Three times; 4. More than three times.
Empirical	3. How advantageous was the use of the VR method in your anatomy education when compared with the conventional methods (2D photos, texts, lectures)?	Linear scale: (very disadvantageous) 1 - 2 - 3 - 4 - 5 (very advantageous)
Empirical	4. With which of the following advantages do you agree?	Multiple answers: 1. Interactivity; 2. Easy to work with; 3. Good visualisation of the models; 4. Possibility to study the models from many points of view; 5. Great variety of the models; 6. Greater motivation and engagement during work; 7. Neither of the listed advantages.
Empirical	5. With which of the following disadvantages do you agree?	Multiple answers: 1. Motion sickness or discomfort while using the VR headset; 2.Technical difficulties with equipment or software; 3. Time-consuming to set up and learn how to use properly; 4. Limited availability of VR headsets; 5. Neither of the listed disadvantages.
Empirical	6. Do you believe that the use of VR facilitated your anatomy education?	Single answer: 1. Yes; 2. No; 3. I am not sure
Empirical	7. How likely is it that you will recommend the use of VR headsets to your fellow medical students?	Linear scale: (very unlikely) 1 - 2 - 3 - 4 - 5 (very likely)
Non-empirical	8. If we have missed a certain advantage, please describe it here:	N.A
Non-empirical	9. If we have missed a certain disadvantage, please describe it here:	N.A
Non-empirical	10. Please describe your main impression of the work with the VR headsets:	N.A
Non-empirical	11. How do you believe the use of VR headsets during neuroanatomy education can be improved?	N.A
Non-empirical	12. Is there anything else you would like to share about your experience using VR headsets for neuroanatomy education?	N.A
